# Behavioural therapy for shift work disorder improves shift workers' sleep, sleepiness and mental health: A pilot randomised control trial

**DOI:** 10.1111/jsr.14162

**Published:** 2024-03-05

**Authors:** Annie Vallières, Alric Pappathomas, Séverine de Billy Garnier, Chantal Mérette, Julie Carrier, Tyna Paquette, Célyne H. Bastien

**Affiliations:** ^1^ École de psychologie Université Laval Québec Québec Canada; ^2^ Centre de recherche CERVO Québec Québec Canada; ^3^ Centre de recherche du Centre hospitalier universitaire de Québec‐Université Laval Québec Québec Canada; ^4^ Département de psychiatrie et de neurosciences, Faculté de médecine Université Laval Québec Québec Canada; ^5^ Département de psychologie Université de Montréal Montréal Québec Canada; ^6^ Centre d'étude avancée en médecine du sommeil, CIUSSS‐NIM Montréal Québec Canada

**Keywords:** behavioural therapy, healthcare, insomnia, shift work disorder, treatment

## Abstract

The present study evaluates the efficacy of behavioural therapy adapted for shift work disorder with a randomised control design in a healthcare population. Forty‐three night shift workers (m. age: 34 years; 77% women) experiencing shift work disorder were randomised to either the behavioural therapy for shift work disorder (BT‐SWD) or a waiting‐list control group offered after the waiting period. Participants completed questionnaires on insomnia, sleepiness and mental health pre‐ and post‐treatment, pre‐ and post‐waiting, and at follow‐up, and a sleep diary. As night shift workers alternate between sleeping during the day after their night shifts and transitioning to nighttime sleep on days off, insomnia severity and sleep variables were analysed for daytime and nighttime sleep. The BT‐SWD involved sleep restriction therapy, stimulus control and fixed sleep periods in the dark. Statistical analyses were performed under intent‐to‐treat and per‐protocol approaches. Repeated‐measures two‐way ANCOVA analysis, controlling for age, sex and pre‐treatment daytime total sleep time, was performed with Bonferroni corrections, and between‐group effect sizes computed. Fourteen participants dropped out after randomisation. Under the intent‐to‐treat analysis, BT‐SWD participants had a significant greater decrease in daytime insomnia severity and an increase in daytime total sleep time at post‐treatment than the control group, with large between‐group effect sizes (−1.25 and 0.89). These corresponding results were also significant with large effect sizes under the per‐protocol analysis. Sleepiness, anxiety and depression levels improved at post‐treatment and maintained at follow‐up when the BT‐SWD treated controls were added to the BT‐SWD group. The behavioural therapy for shift work disorder can be used to improve the sleep and mental health of healthcare night workers.

## 1 INTRODUCTION

Shift workers represent approximately 20%–29% of workers worldwide (IARC Monograph Working Group, 2019; Williams, 2008; Winkler et al., 2018). Shift work involves working outside the conventional hours of 06:00 hours to 18:00 hours, while night shift work involves working at least 3 hours between 23:00 hours and 06:00 hours (Winkler et al., 2018). The most common consequences of shift work are insomnia and sleepiness associated with the work schedule, hence called shift work disorder (SWD; American Academy of Sleep Medicine [AASM], 2014). The prevalence of SWD is estimated at 26.5% among shift workers (Pallesen et al., 2021). SWD has several consequences on the physical and mental health of shift workers, namely elevated anxiety and depressive symptoms (Kalmbach et al., 2015; Reynolds et al., 2022; Torquati et al., 2019; Waage et al., 2014), high risk of gastrointestinal disorders such as gastric ulcers (Knutsson & Boggild, 2010), cardiovascular disorders (Torquati et al., 2018), work absenteeism, as well as low work productivity (Anbazhagan et al., 2016) and satisfaction (Vallières et al., 2021).

Insomnia in shift work aggravates the overall clinical picture of shift workers (Vallières et al., 2014). Shift workers, especially those working on a night shift, often have multiple sleep periods within 24 hours, reverting to nighttime sleep on days off, and they may experience insomnia during any of these sleep periods (Silva‐Costa et al., 2015; Vallières et al., 2021). Shift workers with SWD generally have longer daytime sleep‐onset latency (SOL) and lower objective daytime sleep efficiency (SE) than shift workers without SWD (Vanttola et al., 2019). A complaint of wakefulness during daytime or nighttime sleep characterises shift workers with SWD (Vallières et al., 2021; Vanttola et al., 2019), who also present with more sleepiness before and after a night shift compared with shift workers without SWD (Vanttola et al., 2019).

Most treatments developed to aid the sleep of shift workers are rooted in chronotherapy, pharmacology and naps (Kalkanis et al., 2023). For instance, bright light therapy can be utilised to align the circadian rhythm with the nighttime work schedule and daytime sleep period (Lowden et al., 2019). Hypnotics may be employed to induce and extend daytime sleep, while stimulants like armodafinil, modafinil or caffeine can serve as countermeasures to alleviate sleepiness during wakefulness (Liira et al., 2014). New data suggest that psychological factors are also involved in SWD (Cheng & Drake, 2018), including inaccurate beliefs about sleep, negative work‐related concerns (e.g. fear of making a mistake at work due to sleepiness; Bastille‐Denis et al., 2020) and cognitive activation at bedtime (Vallières et al., 2021), suggesting that cognitive behaviour therapy for insomnia (CBT‐I) could be useful for shift workers. While few studies exist on the efficacy of CBT‐I for shift‐workers, two meta‐analyses and systematic review were conducted to understand treatment effectiveness (Reynolds, Sweetman, et al., 2023; Takano et al., 2023). Reynolds et al. (2023) reviewed eight studies evaluating CBT‐I as the primary intervention where only two were randomised control trials. Takano et al. (2023) included only randomised control trials and found the same two articles. According to these two meta‐analyses, CBT‐I has limited effectiveness to alleviate insomnia in shift workers. The first review showed some significant improvement in shift workers' insomnia severity, sleep quality and SE, but these improvements remain below the clinical threshold known for these variables (Reynolds, Sweetman, et al., 2023). Moreover, both meta‐analyses reported that sleep duration, SOL and wake after sleep onset (WASO) did not significantly improve from baseline to follow‐up.

The lack of improvement in sleep outcomes with CBT‐I for shift workers could be attributed to different factors, such as the need to adapt CBT‐I to the shift work context. Additionally, none of the studies included in the meta‐analyses considered the impact of CBT‐I on daytime and nighttime sleep separately. Vallières and Bastille‐Denis (2012) developed a behavioural treatment for SWD (behavioural therapy [BT]‐SWD) where efficacy was explored in two pilot studies (Claveau et al., 2017; Vallières et al., 2015). BT‐SWD involves sleep restriction therapy (Spielman et al., 1987) and stimulus control (Bootzin, 1972) for insomnia and fixed sleep periods in the dark to help the circadian timing system to be in tune with the sleep and wake cycles (Horowitz et al., 2001). BT‐SWD targets all shift workers' sleep periods (daytime sleep, nighttime sleep, and naps). The two pilot studies, utilising a single‐case design, yielded promising results. They indicated that BT‐SWD can improve daytime and nighttime sleep, reduce mood disturbances (anxiety and depression levels), and reduce the endorsement of dysfunctional beliefs about sleep in night shift workers who work 4–5 nights per week (Claveau et al., 2017; Vallières et al., 2015).

The present study evaluates the effectiveness of BT‐SWD using a randomised control design. It is hypothesised that, compared with a waiting‐list control group, night shift workers with SWD receiving treatment will show improvements in daytime sleep (lower insomnia severity scores, higher sleep time, shorter wake time during sleep, higher SE). As secondary hypotheses, it is expected that, compared with a waiting‐list control group, night shift workers with SWD receiving treatment will exhibit improvements in nighttime sleep (shorter wake time during sleep, higher SE), a decrease in psychological distress (lower depressive and anxiety symptoms), reduced cognitive activation and intrusive thoughts while falling asleep, less endorsement of dysfunctional beliefs about sleep, and greater satisfaction at work.

## 2 METHODS

### 2.1 Design and randomisation

The study followed a randomised controlled trial with a waiting‐list control group design. Participants were randomly assigned under two conditions: the experimental group receiving the BT‐SWD; or the waiting‐list group (control group). A blind statistician programmed with a SAS/STAT PLAN a block randomisation of size four to ensure comparable group sizes after each entry of four new participants. Participants in the control group waited 8 weeks (the length of the treatment) before receiving BT‐SWD. The waiting period was reduced to 1 month for the final year of recruitment due to the high dropout rate during the waiting period. The study was registered retrospectively on 04/06/2019 to ISRCTN where an English summary of the French protocol (which includes two studies) can be found (www.isrctn.com/ISRCTN17016944).

### 2.2 Participants

Inclusion criteria were: (a) age > 18 years; (b) working in the province of Quebec's health network at least 5 nights out of 14 days for at least 3 months; (c) night work had to take place between 24:00 hours and 08:00 hours (± 1 hr) for at least 3 months; (d) be diagnosed with SWD. Criteria (b) and (c) are designed around the commonly used night schedule in the Quebec City area, with the aim of targeting a homogeneous sample of shift workers. Exclusion criteria were: (a) symptoms of a sleep disorder other than SWD (e.g. sleep apnea); (b) major depression with suicidal ideation; (c) psychotic disorder or any disorder resulting from substance abuse; (d) inability to answer questions during interviews or to respond to questionnaires; (e) being visually impaired; (f) having consumed hypnotics more than three times per week in the last month; and (g) consuming more than 10 cups of coffee (or other stimulants) per day. No specific professional activity was targeted. Ethical approval for the multi‐centric project was obtained from the CIUSSS‐CN's (Centre intégré universitaire de santé et de services sociaux Capitale‐Nationale) ethics committee (13‐2016‐131, MP) and approved by the CHU's (Centre hospitalier universitaire) ethics committee (2016‐2810‐CHU de Québec).

Participants were recruited in: (a) hospitals; (b) health and social services centres; (c) long‐term care accommodation centres; and (d) seniors' residences. Participants were recruited through a press conference, through media interviews, and ads posted on various communication channels (e.g. hospitals' social media, bulletin boards).

### 2.3 Measures

#### 2.3.1 Evaluation

After a phone screening to determine participant eligibility, the evaluation consisted of a combination of two semi‐structured interviews. The first one is the Structured Insomnia Interview (SII; Morin, 1993) adapted to screen for SWD and other potential sleep disorders. For more details on the adapted version, see Vallières et al. (2021). The *Mini International Neuropsychiatric Interview* (Sheehan et al., 2006), based on DSM‐IV (American Psychiatric Association [APA], 2000), was used to screen for current and past psychopathologies.

#### 2.3.2 Sleep measures

Measures were carefully chosen to evaluate the sleep patterns of night shift workers, focusing on multiple sleep periods within a 24‐hr period and nighttime sleep on days off. To accomplish this, certain conventional insomnia measures had to be administered twice, specifically addressing nighttime sleep and daytime sleep. Only measures producing primary outcomes were adapted to restrict the multiplication of variables for analysis.

##### Sleep diary

The sleep diary (Lamy et al., 2012), available in both paper and online versions, utilised the questions from the consensus sleep diary (Carney et al., 2012) that participants completed after each sleep period every day. After each sleep period (daytime sleep, nighttime sleep, naps), participants reported what time they went to bed, how long they took to fall asleep, if they woke up, how long they stayed awake, and what time they got out of bed. A nap sleep period refers to any additional sleep period occurring outside of the regular nighttime and daytime sleep periods within a 24‐hr period. From these diaries, total sleep time (TST), total wake time (TWT) and SOL were derived for each participant for daytime sleep, nighttime sleep and napping. The TWT includes early‐morning awakening (EMA), WASO and SOL. EMA is the time spent awake after the last awakening and before getting out of bed. The number of naps per day during days with naps and the nap‐TST were computed. SE was also computed for daytime and nighttime sleep using the respective TST and time in bed of each sleep period. State sleepiness measured by the Stanford sleepiness scale (SSS; Hoddes et al., 1973) was recorded before and after work as well as before and after each sleep period.

#### 2.3.3 Self‐reported measures

Participants completed French versions of self‐reported measures related to SWD symptoms and psychosocial variables. These measures possess adequate psychometric properties, except the Glasgow Content of Thoughts Inventory (GCTI; Harvey & Espie, 2004) and the Work Satisfaction Inventory (Barton et al., 1995) for which the psychometric qualities of the French version have not yet been evaluated.

Insomnia symptoms were evaluated with the Insomnia Severity Index (ISI; Bastien et al., 2001). The total score ranges from “0” to “28”. Scores were classified into four severity categories: no clinically significant insomnia (0–7); subthreshold insomnia (8–14); clinical insomnia of moderate severity (15–21); and severe clinical insomnia (22–28; Bastien et al., 2001). ISI was completed for daytime and nighttime sleep separately. Sleepiness was evaluated by the Epworth Sleepiness Scale (ESS; Johns, 1991). Assessment also included the GCTI (Harvey & Espie, 2004), the Dysfunctional Beliefs and Attitudes about Sleep (DBAS‐16; Morin et al., 2007), the Predisposition Sleep Arousal Scale (PSAS; Nicassio et al., 1985), the Beck Depression Inventory‐II (BDI‐II; Beck et al., 1996), the State–Trait Anxiety Inventory (STAI‐State and STAI‐Trait; Spielberger & Palo Alto, 1983), the Work Satisfaction Scale of the Standard Shiftwork Index (Barton et al., 1995), and the Dyadic Adjustment Scale (DAS; Sabourin et al., 2005).

### 2.4 Treatment

The BT‐SWD involves sleep restriction therapy (Spielman et al., 1987) and stimulus control (Bootzin, 1972) for insomnia and fixed sleep periods in the dark for shift workers (Horowitz et al., 2001). The treatment has been described previously (Vallières & Bastille‐Denis, 2012). BT‐SWD targets all shift workers' sleep periods. Table 1 summarises the treatment content and the approach taken in each session. It included six 30–50‐min individual face‐to‐face sessions conducted at the sleep laboratory. For three participants living outside Quebec City, the treatment was offered online. The first four sessions were given weekly, while the last two were given every 2 weeks. The first session included psychoeducation on sleep, circadian rhythms and SWD, and introduced sleep restriction therapy, while the second session introduced stimulus control. Both sleep restriction and stimulus control therapy were adapted to shift work and needed to be applied in the dark. Subsequent sessions were dedicated to adjusting the sleep windows for the three distinct sleep periods, reviewing information provided during the initial session, and to resolving any problems encountered in the application of the sleep windows. The last session focused also on maintaining therapeutic gains. The application of these procedures was maintained throughout the treatment.

**TABLE 1:** Summary of BT‐SWD components per session.

Sessions	Treatment component
1	Psychoeducation on the circadian rhythm, sleep homeostasis and SWD Introduce target 1: insomnia during nighttime sleep Apply sleep restriction therapy using a night sleep window Apply adapted stimulus control
2	Keep working on target 1 Introduce target 2: insomnia during daytime sleep Apply sleep restriction therapy using a day sleep window Apply adapted stimulus control
3	Keep working on targets 1 and 2 Introduce target 3: nap sleep Apply sleep restriction therapy using a nap sleep window Apply adapted stimulus control Transitions Follow the nap sleep window before starting the first night shift Do not follow the nap sleep window during the transition back to a nocturnal sleep schedule
4 and 5	Keep working on targets 1, 2 and 3
6	Keep working on targets 1, 2 and 3 Maintaining therapeutic gains strategy
General recommendations	Each sleep window is based and adjusted (+15 min, stable, or –15 min) on the average of TST and SE for the related sleep period At each treatment session, sleep windows are adjusted. Problems solving strategies can be applied to encourage participants to follow sleep windows and stimulus control recommendations
Adapted stimulus control for shift workers	Stay in the dark for the entire sleep window duration If awake for more than 20 min, stay in the dark, walk slowly in the bedroom or sit on a chair Return to bed when sleepy or after 20 min of wakefulness

Abbreviations: BT‐SWD, Behavioural therapy for shift work disorder; min, minutes; SE, sleep efficiency; SWD, shift work disorder; TST, total sleep time.

Sleep restriction therapy for insomnia (Spielman et al., 1987) consists of restricting the time in bed to the estimated time asleep using a sleep window that is modified each week depending on the patient's SE. For SWD, sleep restriction therapy was applied similarly but to the three sleep periods in this order: nighttime, daytime, and nap sleep. For the transitional sleep before or after consecutive night shifts, participants were instructed to adhere to their designated day and night sleep windows. Additionally, they were allowed to follow their nap sleep window before commencing the first night shift but not during the transition back to a nocturnal sleep schedule. The participants received a sleep window for each sleep period beginning with nighttime sleep. Each sleep window was based on the TST and SE mean for the related sleep period. For example, in the case of a participant working 9 nights out of 14 days, the initial sleep window for nighttime sleep was determined by the mean night TST (night‐TST) calculated from the 5 nights off during the two pre‐treatment weeks when the participant slept at night. Conversely, the sleep window for daytime sleep was determined based on the average daytime TST (day‐TST) from the preceding week of the treatment session, during which the participant had worked night shifts. Each sleep window was modified following these rules: the length of the sleep window was increased by 15 min if the SE was ≥ 85%. When the SE of the target sleep period (nighttime, daytime or nap) was between 80% and 85%, the sleep window remained stable until the next session. If the effect size (ES) of the target sleep period was < 80%, the sleep window was restricted to the TST of the targeted sleep period. Nighttime and daytime sleep windows could not be lower than 5 hours. For daytime sleep, the time to go to bed was set after returning home.

Stimulus control for insomnia (Bootzin, 1972) consists of six instructions to reinforce the association between the bed and bedroom with sleep and to stabilise sleep. Adapted to shift work, participants were instructed to walk slowly in their bedroom or sit on a chair and stay in the dark when they were awake more than 20 min. They were instructed to return to bed when sleepy or after 20 min of wakefulness.

#### 2.4.1 Treatment adherence

Treatment adherence was assessed using the sleep diary (Agnew et al., 2021) in order to verify whether the participant followed the therapist's recommendations. Each week, the therapist rated whether the participant followed the sleep window for each sleep period. Any deviation of 15 min or less was considered respected. The overall percentage of adherence to the sleep window for each weekly session is the respected wake‐up times and bedtimes divided by the total number of wake‐up times and bedtimes to be respected × 100. From session 3, adherence to stimulus control was also measured. It is computed as the number of times participants adhered to the recommendations versus the number of times they could have done so. The percentage of adherence to all these behavioural procedures (sleep restriction and stimulus control) was then calculated to obtain the overall adherence to each treatment session.

#### 2.4.2 Therapists

Graduate psychology students (six) who had completed their clinical training conducted the evaluation under the supervision of clinical psychologists (first and third authors). One psychologist and four graduate psychology students having experience with CBT‐I and who had completed clinical intervention training offered treatment under the supervision of the first author.

### 2.5 Procedures

After the phone screening, eligible participants were invited to the sleep laboratory at the CERVO Brain Research Centre where they signed the consent form and underwent the evaluation. During the first visit, participants completed self‐reported online questionnaires through PIANO (Portail intégré d'applications numériques pour ordinateur), which is Université Laval's private server. Then, they completed their sleep diaries for 2 weeks. After the 2‐week period, participants came to the sleep laboratory for a second visit. Those who met the inclusion criteria were randomised. Participants randomised for BT‐SWD were instructed to complete a sleep diary throughout the treatment and completed self‐reported questionnaires. Those randomised to the waiting‐list were contacted before the end of the waiting period to complete their post‐waiting sleep diaries and self‐reported questionnaires. They were offered BT‐SWD after their waiting time. Participants who received BT‐SWD were invited to the sleep laboratory for a post‐treatment assessment at the end of treatment. Participants underwent the adapted version of the SII (Morin, 1993) with an independent interviewer, and then completed self‐reported questionnaires. Six months after receiving BT‐SWD, participants were contacted for the completion of self‐reported questionnaires and 2 weeks of sleep diaries.

### 2.6 Assessment periods and outcomes

The BT‐SWD group had three assessment periods lasting 2 weeks: before treatment (pre‐treatment); after treatment (post‐treatment); and 6 months after the end of treatment (follow‐up). The control group had four evaluation periods lasting 2 weeks: before the waiting time (pre‐waiting); after the waiting time (post‐waiting, which became the pre‐treatment for those receiving treatment after the waiting period); after treatment (post‐treatment); and 6 months after the end of treatment (follow‐up).

Remission rates (based on the definition and criteria for remission from DSM‐5; APA, 2013) after treatment, insomnia severity for daytime and nighttime sleep, and daytime sleep variables from the sleep diary were the primary outcomes. Secondary outcome measures included nighttime sleep variables from the sleep diary, sleepiness and psychosocial variables.

### 2.7 Sample size and statistical analyses

Sample size computations were done with the nQuery Advisor software (www.statsol.ie) using our pilot data (Claveau et al., 2017; Vallières et al., 2015). In these two studies, participation rates and attrition rates were about 90% and 15%. Allocating 22 participants to each group (BT‐SWD and control) ensured 80% statistical power, enabling the detection of a significant difference between the BT‐SWD group (pre‐ to post‐treatment) and the control group (pre‐ to post‐waiting) corresponding to an ES of 0.76, with a unilateral alpha set at 5%. A unilateral alpha was chosen because it was expected that the BT‐SWD group would have a larger improvement than the control group. For the treatment stability over a 6‐month follow‐up, we considered adding the participants from the control group who would accept to receive BT‐SWD after the waiting period. Thus, it would increase the sample size up to a maximum of 44 participants, leading to a statistical power of 85%, which would detect a significant time effect, corresponding to an ES of 0.50 with a unilateral test of 0.013 (= 5% ÷ 4).

Statistical analyses were conducted with SAS/STAT software, version 9.4 for Windows (SAS Institute, 2005). Descriptive statistics of sociodemographic and pre‐treatment outcome variables were computed to describe the whole sample, the experimental (BT‐SWD) and the control (waiting‐list) groups. Participants in the treatment and control groups were compared using *t*‐tests for continuous variables or Mann–Whitney tests (non‐parametric tests) when needed, and Fisher's exact tests for categorical variables. These analyses were conducted to verify that randomisation resulted in equivalent groups. If one of the pre‐treatment sleep outcome variables differed between groups with an alpha level of 0.05, it was added as a covariable, with age and sex, in the ANCOVA (Analysis of Covariance) described below. Pre‐treatment ISI scores of participants who completed the experiment (BT‐SWD and control group) were compared with those who dropped out to ensure that insomnia severity does not explain treatment participation and efficacy. Descriptive analyses were conducted on treatment adherence based on the sleep diary.

The statistical analyses were divided into two parts. The first one compared the treatment and the control groups pre‐ and post‐treatment. Because the control group was offered to receive the treatment after the waiting period (i.e. before follow‐up), there was no additional control assessment time following the post‐treatment. Therefore, the second part of the analysis focused on assessing the stability over time for all participants who received the treatment, involving three assessment points (pre‐, post‐, follow‐up).

For the first part, the primary and secondary outcomes were analysed with the two complementary strategies recommended by Tripepi et al. (2020), i.e. the intent‐to‐treat (ITT) and per‐protocol (PP) analyses. In the ITT approach, all workers who were randomised were included in the analysis, and the last observation carried forward (LOCF) method was used to impute missing data. The LOCF consisted of imputing the pre‐treatment observation as the post‐treatment missing value, thus creating a stability of the severity of the symptoms of insomnia over time (from pre‐ to post‐treatment). Therefore, LOCF is a conservative approach for the evaluation of the treatment effect if the data are missing completely at random (Zhang & Thorburn, 2022) or if missing data occurred more often in the control group, especially when subjects tended to worsen over time without treatment. According to Tripepi et al. (2020), the ITT approach evaluates the study efficacy. Owing to attrition occurring mainly before the treatment initiation, data were thereafter also analysed with a PP strategy that intended to analyse data from participants who strictly adhered to the study protocol, thus evaluating the treatment efficacy among those who completed the study while avoiding imputation (Tripepi et al., 2020). For both the ITT and the PP strategies, the MIXED procedure of SAS was carried out to handle fixed and random effects with balanced or unbalanced data, as described below.

For primary and secondary outcomes, a repeated‐measures two‐way ANCOVA analysis using the MIXED procedure of SAS was performed in which the “Group” (treatment versus control) and “Time” (pre‐ and post‐treatment or pre‐ and post‐waiting in the control group) were the two main factors, and in which the Group by Time interaction term was included to test the difference in change in the dependent variables over time between groups. Sex and age measures were covariables. The significance of the Group by Time interaction was verified to see if change in dependent variables over time (from pre‐ to post‐treatment) was stronger in the treatment than in the control group, thus revealing the treatment efficacy. The ES of the efficacy was assessed by computing the standardised difference between groups in their respective change from baseline.

For the second part of the analysis, the stability of treatment efficacy over 6 months was assessed by pooling the BT‐SWD with the waiting control group, and performing a one‐way repeated‐measures ANCOVA analysis with “Time” as the main factor, going from pre‐treatment to post‐ and 6‐month follow‐up. Only the PP strategy was used to evaluate the stability over time. If the “Time” effect was significant, a post hoc analysis compared the gains at post‐treatment and at the 6‐month follow‐up, and the corresponding standardised ES were assessed.

Bonferroni correction was applied per category. Adjusting alpha for daytime sleep is 0.01 (0.05/5), for nighttime sleep is 0.01 (0.05/5), for insomnia severity and sleepiness is 0.017 (0.05/3), for psychological variables is 0.007 (0.05/7), and for social variables is 0.025 (0.05/2). The assumptions of normality and homogeneity of variance underlying the ANCOVA models were tested by examining the distribution of the residuals using the normal probability plot, the measures of Skewness and Kurtosis as well as the Shapiro–Wilk and the Kolmogorov–Smirnov tests.

## 3 RESULTS

### 3.1 Participants flowchart

**FIGURE 1:**
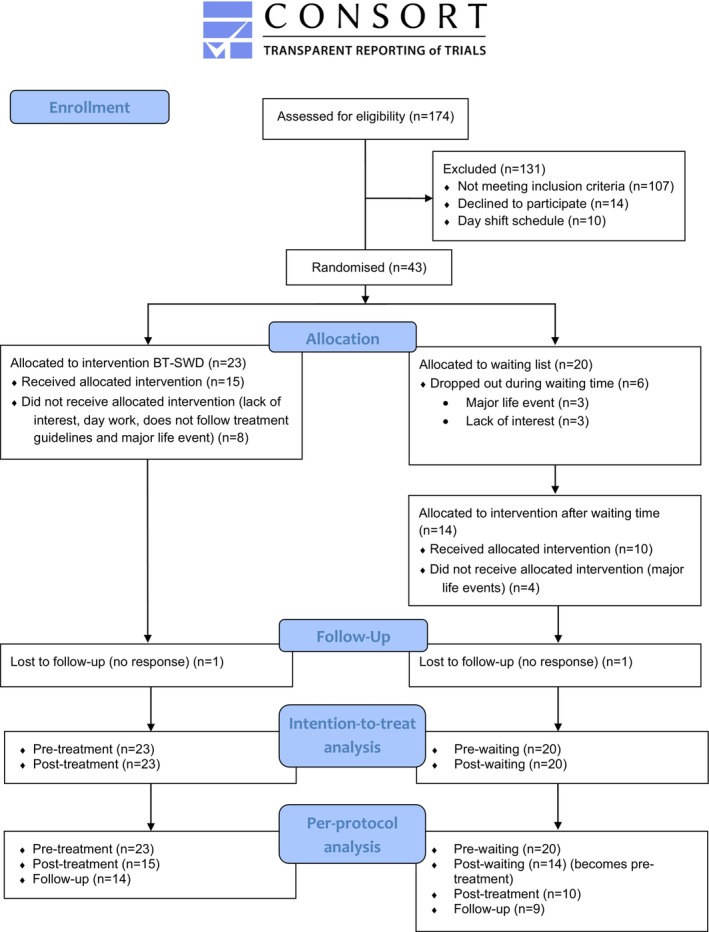
Participants' flowchart.

Participants were recruited between 2016 and 2020 until the beginning of the COVID‐19 pandemic. Figure 1 presents the participants' flowchart. Potential participants (*n* = 174) completed a phone screening interview for eligibility assessment. Of these, 96 were eligible and completed the evaluation, resulting in the diagnosis of SWD in 67 participants. Among them, 24 refused to receive treatment either because they were not interested or because their work schedule changed to a day shift. Therefore, 43 participants were randomised into BT‐SWD (*n* = 23) or a waiting‐list control (*n* = 20) group. Six participants dropped out while waiting and four more did so when treatment started after the waiting period. Two participants from the waiting group had a waiting time period of 4 weeks. One of them initiated the treatment after the waiting period but dropped out during week 5 of the treatment. Eight participants from the treatment group dropped out before the beginning of treatment. The main reasons included a major life event, a lack of interest, or return to a day work schedule. Overall, 25 participants completed the treatment: 15 from the BT‐SWD group; and 10 from the waiting‐list control group. Twenty‐three completed the 6‐month follow‐up: 14 in the BT‐SWD group and nine in the control group.

### 3.2 Sample description

Table 2 shows the sociodemographic variables and sleep variables at pre‐treatment. The mean age of the sample was 34 years (SD = 9.0) and 77% were women. Eighty‐eight percent of the sample were nurses or beneficiary attendants/orderlies, and 12% were other hospital workers such as housekeeping and security employees. Participants worked on average 8.58 (SD = 1.10) night shifts of an 8‐hour duration per 14 days, and had an average of 5.20 (SD = 0.10) days off per 14 days. No participant worked evening shifts and only two worked day shifts in rotation with night shifts. Twenty‐three participants (13 in BT‐SWD and 10 in the control group) at pre‐treatment and eight at post‐treatment were taking a hypnotic medication, an anti‐psychotic medication or melatonin on an irregular basis. Regarding psychiatric conditions, 32.6% of participants presented with an anxiety disorder, a major depressive disorder or both. There was no group difference regarding sociodemographic variables. However, groups were different at pre‐treatment on day‐TST (*p* = 0.04).

**TABLE 2:** Comparison of the BT‐SWD and Control groups on the sociodemographic and pre‐treatment sleep variables.

	All *n* = 43	BT‐SWD *n* = 23	Control *n* = 20	*p*‐Value^a^
Sociodemographic variables				
Age in years (M, SD)	33.5 (9.0)	35.3 (9.3)	31.5 (8.5)	0.17
Gender (% of women)	76.7	87.0	65.0	0.15
Years of schooling (M, SD)	15.9 (3.5)	15.7 (4.5)	16.2 (1.7)	0.58
Civil status (%)				0.53
Single	37.2	30.4	45.0	
In couple/married	60.5	65.2	55.0	
Divorced	2.3	4.4	0.0	
Nationality (% Canadian)	90.7	82.6	100.0	0.11
Full‐time employment (%)	79.1	78.3	80.0	1.00
Annual income (%)				0.52
< $20,000	2.3	4.4	0.0	
$20,000–35,000	0.0	0.0	0.0	
$35,000–50,000	37.2	34.8	40.0	
$50,000–65,000	32.6	26.1	40.0	
> $65,000	27.9	34.8	20.0	
Type of work (%)				0.13
Nurse	78.1	66.7	90.0	
Beneficiary attendant/orderly	9.8	19.1	0.0	
Other	12.2	14.3	10.0	
Years working under this schedule (M, SD)	4.8 (5.3)	4.4 (6.1)	5.3 (4.5)	0.71
Years suffering of sleep difficulties (Median, IQR)	3.5 (4.2)	2.5 (3.7)	4.5 (7)	0.05
Number of night shifts^b^ on 14‐day work schedule (Median, IQR)	9 (1)	9 (1)	9 (1.75)	0.51
Number of days off on 14‐day work schedule (Median, IQR)	5 (1)	5 (1)	5 (1.75)	0.07
Current comorbid diagnosis (%)				0.20
None	67.4	56.5	80.0	
Major depressive disorder	14.0	21.7	5.0	
Anxiety disorder	14.0	13.0	15.0	
Major depressive and anxiety disorder	4.7	8.7	0.0	
Insomnia severity				
ISI‐day	13.8 (4.5)	14.5 (4.0)	12.9 (4.9)	0.24
ISI‐night	9.7 (5.5)	9.9 (4.9)	9.6 (6.3)	0.85
Sleep variables				
Daytime sleep				
TST (min)	342.6 (91.2)	316.7 (74.3)	373.9 (101.5)	**0.04**
TWT (min)	49.2 (30.9)	46.6 (29.5)	52.3 (33.1)	0.56
SOL (min)	16.9 (14.5)	18.8 (18.3)	14.5 (7.7)	0.32
SE (%)	87.5 (7.0)	87.2 (7.7)	87.9 (6.2)	0.77
Nighttime sleep				
TST (min)	455.8 (84.0)	441.4 (90.0)	473.1 (74.7)	0.23
TWT (min)	57.0 (36.4)	51.7 (32.9)	63.5 (40.2)	0.30
SOL (min)	16.0 (14.6)	16.4 (17.3)	15.5 (11.0)	0.85
SE (%)	89.0 (7.0)	89.8 (6.6)	88.1 (7.4)	0.43

*Note*: Bold indicates the significant results.

Abbreviations: BT‐SWD, behavioural therapy for shift work disorder; ISI‐day, Insomnia Severity Index for daytime sleep; ISI‐night, Insomnia Severity Index for nighttime sleep; IQR, interquartile range; M, mean; SD, standard deviation; SE, sleep efficiency; SOL, sleep‐onset latency; TST, total sleep time; TWT, total wake time.

^a^ *p*‐Values for comparing BT‐SWD and Control groups are from *t*‐test or Mann–Whitney test for continuous variables, and from Fisher's exact test for contingency tables.

^b^ Shifts durations are 8 hr. Night shift is from 24:00 hours to 08:00 hours. Day shift is from 08:00 hours to 16:00 hours.

### 3.3 Treatment attrition and adherence

The attrition rate was 32.6% (14/43), from which eight participants were in the BT‐SWD group and six in the control group. In the BT‐SWD group, participants who dropped out (*n* = 8) were not significantly different from those who received BT‐SWD (*n* = 15) nor were those in the control group (*n* = 20) in terms of insomnia severity for daytime and nighttime sleep (*F*_2,40_ = 2.19 and 0.17, respectively, *p* = 0.13 and 0.85). Participants receiving treatment attended all treatment sessions. The percentage of adherence to all behavioural procedures combined increased with each session. The percentage fluctuated, starting at 64% during the first week of treatment, increasing to 81% in the third week, and reaching 82% for the final 2 weeks of treatment.

### 3.4 Primary outcomes

#### 3.4.1 Remission rates

At post‐treatment, 24 participants completed the SII‐adapted. Of these, 92% of participants (*n* = 22/24) were no longer diagnosed with SWD. All participants who received treatment were in remission from SWD (11 = partial; 13 = complete).

#### 3.4.2 Insomnia severity

**FIGURE 2:**
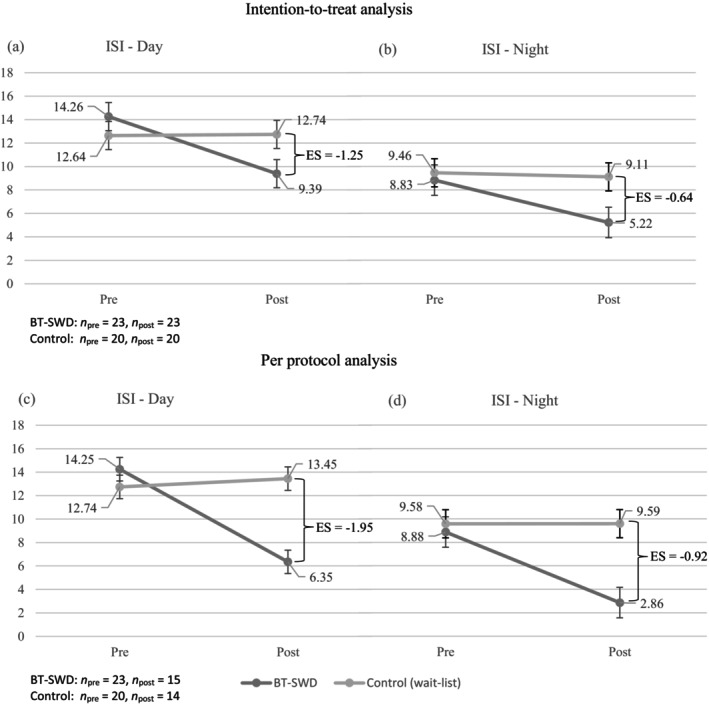
ISI for daytime and nighttime sleep according to group (BT‐SWD and Control) and time (pre‐ and post‐treatment for BT‐SWD, and pre‐ and post‐waiting for the control group). Group by Time interactions are significant for ISI‐day with ITT analysis (*p* = 0.0002), and for both ISI‐day and ‐night with PP analysis (*p* < 0.0001 and 0.006). BT‐SWD, behavioural treatment for shift work disorder; ISI, Insomnia Severity Index; ITT, intent‐to‐treat; PP, per‐protocol; Pre, pre‐treatment.

Figure 2 presents ISI scores and between‐group ESs for daytime and nighttime sleep using both the ITT analysis and the PP analysis, categorised by group (BT‐SWD or Control). Under the ITT analysis approach with a Bonferroni correction, a Group by Time interaction was significant for daytime insomnia severity (*F*_1,41_ = 16.65, *p* = 0.0002). The BT‐SWD group had a significant greater decrease in daytime insomnia severity at post‐treatment than the control group with a between‐group ES of −1.25. It is noteworthy that the corresponding result, derived from the PP analysis, was also significant (*F*_1,27_ = 40.99, *p* < 0.0001), with a between‐group ES of −1.95. This indicates a substantial difference of almost two standard deviations in efficacy between the pre‐ and post‐treatment groups. Furthermore, also based on the PP analysis, a Group by Time interaction for the nighttime insomnia severity was detected (*F*_1,27_ = 9.04, *p =* 0.0057), with a between‐group ES of −0.92.

#### 3.4.3 Daytime sleep variables

Table 3 presents adjusted means, standard errors and between‐group ESs for TST, TWT, SOL and SE during daytime and nighttime sleep, as well as nap‐TST using both the ITT and the PP approaches, categorised by group (BT‐SWD or Control). Under the ITT analysis with a Bonferroni correction, a significant Group by Time interaction was detected for day‐TST (*F*_1,24_ = 8.52, *p* = 0.008), with a between‐group ES of 0.89. The corresponding results, derived from the PP approach, were also significant (*F*_1,27_ = 9.78, *p* = 0.005), with a between‐group ES of 0.96. Additionally, it is noteworthy that the Group by Time interaction for day‐SOL was not significant under the Bonferroni correction (0.05/5 = 0.01; *F*_1,23_ = 5.91, *p* = 0.02). However, given the *p*‐value observed and the large between‐group ES (−0.75), day‐SOL must be monitored in other studies.

**TABLE 3:** Adj M and SEs for sleep variables according to group (BT‐SWD or Control) and time (pre‐, post‐treatment; pre‐, post‐waiting) from the results of the repeated measures ANCOVA analyses adjusted for age and sex.

					ITT analysis^b^		PP analysis^c^	
	BT‐SWD		Control		ANCOVA	ES	ANCOVA	ES
Sleep variables	Pre‐tx (*n* = 23) Adj M (SE)	Post‐tx (*n* = 23) Adj M (SE)	Pre‐wait (*n* = 20) Adj M (SE)	Post‐wait (*n* = 20) Adj M (SE)	*F* (*p*_interaction_)	Between groups	*F* (*p*_interaction_)	Between groups
Daytime sleep								
Day‐TST^a^	336.4 (7.5)	372.8 (9.2)	346.3 (7.5)	333.2 (9.4)	**8.52 (0.008)**	0.89	**9.78 (0.005)**	0.96
Day‐TWT	49.4 (6.9)	30.6 (6.9)	49.5 (6.6)	39.2 (6.6)	1.03 (0.32)	−0.31	1.81 (0.19)	−0.41
Day‐SOL	19.4 (3.5)	12.0 (3.5)	14.7 (3.3)	13.8 (3.3)	4.31 (0.04)	−0.63	5.91 (0.02)	−0.75
Day‐SE	86.5 (1.6)	91.8 (1.6)	88.1 (1.5)	90.1 (1.5)	2.48 (0.12)	0.48	4.08 (0.06)	0.62
Sleepiness upon rising	3.5 (0.2)	3.3 (0.2)	3.6 (0.2)	3.6 (0.2)	0.61 (0.44)	−0.24	2.81 (0.11)	−0.54
Nighttime sleep								
Night‐TST	435.1 (18.9)	441.0 (18.9)	467.0 (17.9)	471.8 (17.9)	0.00 (0.95)	0.02	0.04 (0.85)	0.06
Night‐TWT	50.1 (9.0)	29.7 (9.0)	63.5 (8.5)	59.4 (8.5)	4.19 (0.05)	−0.63	5.14 (0.03)	−0.70
Night‐SOL	15.4 (3.6)	9.0 (3.6)	15.1 (3.4)	14.8 (3.4)	4.86 (0.03)	−0.67	6.24 (0.02)	−0.77
Night‐SE	90.0 (1.7)	94.2 (1.7)	88.1 (1.6)	89.0 (1.6)	4.34 (0.04)	0.64	4.85 (0.04)	0.68
Sleepiness upon rising	3.0 (0.2)	2.8 (0.2)	3.1 (0.2)	3.0 (0.2)	0.38 (0.54)	−0.19	1.73 (0.20)	−0.43
Nap								
TST	76.5 (9.5)	77.4 (9.5)	73.2 (8.9)	71.7 (8.9)	0.11 (0.74)	0.10	0.16 (0.69)	0.13

*Note*: Bonferroni correction applied for daytime and nighttime sleep, adjusting alpha to 0.01 (0.05/5). Significant results are in bold.

Abbreviations: Adj M, adjustment mean; BT‐SWD, behavioural therapy for shift work disorder; ES between groups, effect size of the difference in the mean change from pre‐treatment between groups; ITT, intent‐to‐treat; Post‐tx, post‐treatment; Post‐wait, post‐waiting time; PP, per‐protocol; Pre‐tx, pre‐treatment; Pre‐wait, pre‐waiting time; SE, sleep efficiency; SE, standard error; SOL, sleep‐onset latency; TST, total sleep time; TWT, total wake time.

^a^ Because the two groups differ on pre‐treatment day‐TST (Table 2), it was added as covariable in the ANCOVA.

^b^ *n* = 23 in the BT‐SWD group for all measures except for nap sleep (*n* = 22) and *n* = 20 in the Control group for all measures.

^c^ *n* = 23 in the BT‐SWD group at pre‐treatment and *n* = 15 at post‐treatment, and *n* = 20 in the Control group at pre‐waiting and *n* = 14 at post‐waiting.

### 3.5 Secondary outcomes

#### 3.5.1 Nighttime sleep variables and nap TST

No significant Group by Time interaction was detected for nighttime sleep variables under the ITT or the PP analysis approaches with a Bonferroni correction. However, with the PP strategy, the Group by Time interactions were nearly significant for night‐TWT, night‐SOL and night‐SE (*p* = 0.03, 0.02 and 0.04, respectively), with corresponding between‐group ESs of −0.70, −0.77 and 0.68, respectively, suggesting that these three nighttime variables must be monitored in other studies. Moreover, it is important to note that participants slept about 75 min (adjusted means range: 69.19–78.82 min) while napping an average of 1.21 times per day (based only on days with naps) at pre‐treatment and 1.18 times per day at post‐treatment.

#### 3.5.2 Self‐reported sleepiness and psychosocial variables

Table 4 shows the adjusted means, standard error and between‐group ESs for sleepiness and psychosocial variables according to group using both the ITT and PP approaches, categorised by group (BT‐SWD or Control). With an ITT approach and a Bonferroni correction, a significant Group by Time interaction was detected for intrusive thoughts before bedtime (*F*_1,41_ = 12.82, *p* = 0.0009), with a between‐group ES of −1.09. The corresponding result is also significant from the PP approach (ES = −1.53). Under the same PP approach, significant Group by Time interactions were detected for cognitive arousal and trait anxiety (*F*_1,27_ = 8.6 and 8.7, *p* = 0.007), and with between‐group ESs of −0.89 and − 0.90, respectively. For DBAS and depression symptoms, the Group by Time interactions were nearly significant (*p* = 0.01), with between‐group ESs of −0.81 and −0.85, respectively, and must be monitored on other studies.

**TABLE 4:** Adj M and SE for sleepiness and psychosocial variables according to group (BT‐SWD or Control) and time (pre‐, post‐treatment or pre‐, post‐waiting).

					ITT analysis^a^		PP analysis^b^	
Variables	BT‐SWD		Control		ANCOVA	ES	ANCOVA	ES
	Pre‐tx (*n* = 23) Adj M (SE)	Post‐tx (*n* = 23) Adj M (SE)	Pre‐wait (*n* = 20) Adj M (SE)	Post‐wait (*n* = 20) Adj M (SE)	*F* (*p*_interaction_)	Between groups	*F* (*p*_interaction_)	Between groups
Sleepiness								
Sleepiness (ESS)	10.6 (1.1)	9.0 (1.1)	9.5 (1.0)	8.7 (1.0)	1.24 (0.27)	−0.34	1.47 (0.24)	−0.37
Psychological								
Intrusive thoughts (GCTI)	52.3 (3.1)	41.4 (3.1)	49.8 (2.9)	49.3 (2.9)	**12.82 (0.0009)**	−1.09	**25.09 (< 0.0001)**	−1.53
Beliefs (DBAS‐16)	4.8 (0.3)	4.0 (0.3)	4.7 (0.3)	4.6 (0.3)	4.66 (0.04)	−0.66	6.99 (0.01)	−0.81
Cognitive activation (PSAS‐cognitive)	17.5 (1.5)	14.0 (1.5)	18.2 (1.4)	17.3 (1.4)	5.01 (0.03)	−0.68	**8.60 (0.007)**	−0.89
Physiological activation (PSAS‐physiological)	12.4 (1.0)	10.7 (1.0)	12.5 (0.9)	12.3 (0.9)	2.13 (0.15)	−0.45	3.40 (0.08)	−0.56
Anxiety (trait)	38.7 (2.4)	32.9 (2.4)	36.8 (2.3)	35.5 (2.3)	4.92 (0.03)	−0.68	**8.69 (0.007)**	−0.90
Anxiety (state)	35.2 (2.4)	32.3 (2.4)	33.2 (2.3)	32.4 (2.3)	1.11 (0.30)	−0.32	2.50 (0.13)	−0.48
Depression (BDI‐II)	12.1 (1.9)	6.9 (1.9)	9.8 (1.8)	7.9 (1.8)	3.78 (0.06)	−0.59	7.67 (0.01)	−0.85
Social								
Work satisfaction	26.5 (0.9)	26.8 (0.9)	28.0 (0.9)	27.7 (0.9)	0.34 (0.56)	0.18	0.35 (0.56)	0.18
Marital distress (DAS‐16)	74.4 (2.1)	77.6 (2.1)	74.5 (1.9)	75.7 (1.9)	0.93 (0.34)	0.33	0.72 (0.41)	0.29

*Note*: Bonferroni correction applied, per category. Adjusting alpha for psychological variables is 0.007 (0.05/7) and for social variables is 0.025 (0.05/2). Significant results are in bold.

Abbreviations: Adj M, adjustment mean; Anxiety STATE, State–Trait Anxiety Inventory‐State; Anxiety TRAIT, State–Trait Anxiety Inventory‐Trait; BDI‐II, Beck Depression Inventory‐II; BT‐SWD, behavioural therapy for shift work disorder; DAS‐16, Dyadic Adjustment Scale 16‐item; DBAS‐16, Dysfunctional Beliefs and Attitudes about Sleep scale 16‐item; ES between groups, effect size of the difference in the mean change from pre‐treatment between groups; ESS, Epworth Sleepiness Scale; GCTI, Glasgow Content of Thoughts Inventory; ITT, intent‐to‐treat; Post‐tx, post‐treatment; Post‐wait, post‐waiting time; PP, per‐protocol; Pre‐tx, pre‐treatment; Pre‐wait, pre‐waiting time; PSAS‐cognitive, Predisposition Sleep Arousal Scale cognitive scale; PSAS‐physiological, Predisposition Sleep Arousal Scale physiology subscale; SE, standard error.

^a^ *n* = 23 in the BT‐SWD group for all measures except for DAS‐16 (*n* = 18), and *n* = 20 in the Control group for all measures except for DAS‐16 (*n* = 17).

^b^ *n* = 23 in the BT‐SWD group at pre‐treatment and *n* = 15 at post‐treatment, and *n* = 20 in the Control group at pre‐waiting and *n* = 14 at post‐waiting.

#### 3.5.3 Stability of treatment efficacy

Table 5 shows the adjusted means, standard errors and ESs for the effect of time for daytime and nighttime sleep, insomnia severity, sleepiness, and psychosocial variables for pre‐, post‐treatment and follow‐up of the overall sample receiving BT‐SWD (including the control group after waiting). Significant time effects were observed for day‐TWT and day‐SE (*F*_2,37_ = 12.2, and 14.2, respectively, *p* ≤ 0.0001). ESs showed that improvements were between pre‐ and post‐treatment (ESs time = −1.02, 1.10, respectively, *p* = 0.0001), and maintained at follow‐up (ESs time = 0.12 and − 0.09, *p* = 0.62 and 0.69). For nighttime sleep, time effects were observed for night‐TWT, night‐SOL and night‐SE (*F*_2,39_ = 8.8, 4.7 and 10.5, respectively, *p* = 0.0007, 0.01 and 0.0002). ESs for time effects showed that improvements were between pre‐ and post‐treatment (ESs time = −0.92, −0.57, and 1.00, respectively, *p* = 0.0002, 0.01 and 0.0001), and maintained at follow‐up (ESs time = 0.14, −0.14 and − 0.16, *p* = 0.54, 0.54 and 0.48).

**TABLE 5:** Adj M and SEs for the whole sample receiving BT‐SWD at pre‐, post‐treatment and follow‐up.

Variables	Assessment times			ANCOVA repeated‐measures	Post‐tx versus pre‐tx	Follow‐up versus post‐tx
	Pre‐tx (*n* = 33^a^) Adj M (SE)	Post‐tx (*n* = 25^b^) Adj M (SE)	Follow‐up (*n* = 23^c^) Adj M (SE)	*F* time (*p*)	ES time (*p*)^d^	ES time (*p*)^d^
Daytime sleep						
Day‐TST	326.7 (12.6)	362.4 (15.1)	344.9 (14.4)	3.1 (0.06)		
Day‐TWT	48.3 (4.5)	19.8 (5.7)	23.3 (5.3)	**12.2 (0.0001)**	−1.02 (0.0001)	0.12 (0.62)
Day‐SOL	20.0 (2.9)	10.1 (3.7)	10.5 (23.4)	3.9 (0.03)	−0.54 (0.03)	0.02 (0.93)
Day‐SE	87.2 (1.2)	95.0 (1.5)	94.3 (1.4)	**14.2 (< 0.0001)**	1.10 (0.0001)	−0.09 (0.69)
Sleepiness upon rising	3.6 (0.2)	3.3 (0.2)	3.8 (0.2)	1.5 (0.23)		
Nighttime sleep						
Night‐TST	446.4 (12.8)	454.6 (14.5)	480.4 (14.5)	2.8 (0.07)		
Night‐TWT	52.9 (7.0)	24.0 (7.9)	28.7 (7.9)	**8.8 (0.0007)**	−0.92 (0.0002)	0.14 (0.54)
Night‐SOL	15.5 (2.8)	8.2 (3.1)	6.3 (3.1)	**4.7 (0.01)**	−0.57 (0.01)	−0.14 (0.54)
Night‐SE	89.6 (1.3)	95.4 (1.5)	94.4 (1.5)	**10.5 (0.0002)**	1.00 (0.0001)	−0.16 (0.48)
Sleepiness upon rising	3.2 (0.2)	2.9 (0.2)	2.9 (0.2)	1.0 (0.37)		
Insomnia severity and sleepiness						
ISI‐day	14.2 (0.8)	7.0 (0.9)	6.6 (0.9)	**47.0 (< 0.0001)**	−1.84 (0.0001)	−0.10 (0.63)
ISI‐night	10.1 (0.9)	4.6 (1.0)	7.2 (1.1)	**16.8 (< 0.0001)**	−1.15 (0.0001)	0.53 (0.01)
Sleepiness (ESS)	10.2 (0.9)	7.9 (0.9)	8.6 (1.0)	**10.6 (0.0002)**	−0.90 (0.0001)	0.27 (0.20)
Psychological variables						
Intrusive thoughts (GCTI)	52.7 (2.2)	39.1 (2.3)	42.0 (2.4)	**23.7 (< 0.0001)**	−1.38 (0.0001)	0.29 (0.16)
Beliefs (DBAS‐16)	4.8 (0.2)	3.8 (0.2)	3.9 (0.3)	**14.4** (**< 0.0001)**	−1.06 (0.0001)	0.06 (0.78)
Cognitive activation (PSAS cognitive)	18.3 (1.1)	13.3 (1.2)	13.7 (1.2)	**17.0 (< 0.0001)**	−1.16 (0.0001)	0.08 (0.69)
Physiological activation (PSAS physiological)	12.6 (0.7)	10.0 (0.8)	11.0 (0.8)	**8.4 (0.0008)**	−0.81 (0.0002)	0.29 (0.17)
Anxiety (trait)	39.5 (2.0)	32.8 (2.1)	33.7 (2.2)	**9.6 (0.0003)**	−0.87 (0.0001)	0.11 (0.59)
Anxiety (state)	35.1 (2.0)	29.9 (2.1)	31.8 (2.2)	**6.0 (0.0047)**	−0.69 (0.0013)	0.24 (0.25)
Depression (BDI‐II)	12.0 (1.4)	4.9 (1.5)	7.4 (1.6)	**13.8 (< 0.0001)**	−1.05 (0.0001)	0.37 (0.08)
Social variables						
Work satisfaction	27.1 (0.9)	26.1 (0.9)	24.4 (1.0)	**5.0 (0.01)**	−0.06 (0.76)	−0.62 (0.005)
Marital distress (DAS‐16)	75.0 (1.8)	77.4 (1.9)	76.2 (2.1)	1.4 (0.25)		

*Note*: Bonferroni correction is applied per category. Adjusting alpha for daytime sleep is 0.01 (0.05/5), for nighttime sleep is 0.01 (0.05/5), for insomnia severity and sleepiness is 0.017 (0.05/3), for psychological variables is 0.007 (0.05/7), and for social variables is 0.025 (0.05/2). Significant results are in bold.

Abbreviations: Adj M, adjustment mean; Anxiety STATE, State–Trait Anxiety Inventory‐State; Anxiety TRAIT, State–Trait Anxiety Inventory‐Trait; BDI‐II, Beck Depression Inventory‐II; BT‐SWD, Behavioural Therapy for Shift Work Disorder; DAS‐16, Dyadic Adjustment Scale 16‐item; DBAS‐16, Dysfunctional Beliefs and Attitudes about Sleep scale 16‐item; ES time, effect size for time effect; ESS, Epworth Sleepiness Scale; GCTI, Glasgow Content of Thoughts Inventory; ISI, Insomnia Severity Index; Post‐tx, post‐treatment; Pre‐tx, pre‐treatment; PSAS‐cognitive, Pre‐Sleep Arousal Scale cognitive scale; PSAS‐physiological, Pre‐Sleep Arousal Scale physiology subscale; SE, sleep efficiency; SE, standard error; SOL, sleep‐onset latency; TST, total sleep time; TWT, total wake time.

^a^ *n* = 33 except for DAS‐16 (*n* = 26), for daytime and nighttime sleep (*n* = 32), for sleepiness upon rising (*n* = 28), and nap sleep (*n* = 30).

^b^ *n* = 25 except for DAS‐16 and nighttime sleep (*n* = 20), daytime sleep (*n* = 18), and nap sleep (*n* = 19).

^c^ *n* = 23 except for DAS‐16 (*n* = 17), for cognitive activation (*n* = 22), for daytime and nighttime sleep (*n* = 22), for sleepiness upon rising (n = 21), and nap sleep (*n* = 20).

^d^ Post hoc analyses were computed only if the “Time” effect was significant.

For daytime and nighttime insomnia severity and sleepiness, significant time effects were observed (*F*_2,46_ = 47.0, 16.8, and 10.6, respectively, *p* < 0.0001, < 0.0001 and = 0.0002). ESs for time effects showed that improvements were between pre‐ and post‐treatment (ESs time = −1.84, −1.15 and −0.90, respectively), and maintained at follow‐up (ESs time = −0.10, 0.53 and 0.27, *p* = 0.63, 0.01 and 0.20). Time effects were significant for all psychological variables with large time effects from pre‐ to post‐treatment (ESs post–pre‐ = −1.38, −1.06, −1.16, −0.81, −0.87, −0.69 and − 1.05, respectively, *p* = 0.0001, 0.0001, 0.0001, 0.0002, 0.0001, 0.0013 and 0.0001, respectively), and were all maintained at the 6‐month follow‐up (ESs post‐ to follow‐up = 0.29, 0.06, 0.08, 0.29, 0.11, 0.24, 0.37, respectively, *p* = 0.16, 0.78, 0.69, 0.17, 0.59, 0.25 and 0.08). For social variables, work satisfaction presented a decrease from post‐treatment to follow‐up, indicating that participants had less job satisfaction at that time (ES = −0.62, *p* = 0.005).

## 4 DISCUSSION

The present study provides evidence that BT‐SWD is effective in improving night shift workers' daytime insomnia severity, daytime TST and intrusive thoughts before bedtime from both ITT and PP analysis strategies. Furthermore, the PP approach shows that BT‐SWD is effective for decreasing cognitive arousal at bedtime and trait anxiety. Importantly, all participants were in partial or complete remission from SWD after treatment. Further improvements are observed on daytime and nighttime insomnia severity, sleepiness, daytime and nighttime SE and wake time, anxiety, depression and beliefs about sleep when the sample size is increased with participants who received treatment after the waiting period. Moreover, most of these improvements are stable over time. Shift workers who received BT‐SWD greatly adhered to treatment procedures and attended all treatment sessions. This study is the first to evaluate the efficacy of an adapted behavioural treatment for night shift workers, considering all sleep periods and compared with a control group.

It is important to note the high attrition rate of the present study. Other studies on CBT‐I and shift workers found a similarly high attrition rate, frequently exceeding 20% (Reynolds, Kyle, et al., 2023). Therefore, the attrition rate provides valuable insights into the unique characteristics of the night shift worker population. The majority of attrition cases occurred before the treatment began, primarily due to the logistical challenges faced by this specific population. From this study's experience, 14–22 weeks of clinical experimentation for a population of night shift workers leads to attrition. They discontinued participation either due to vacation plans, the availability of transitioning to a day schedule, or the complexity of attending face‐to‐face treatment sessions at the laboratory while managing their work schedules. Consequently, future studies might consider online and shorter interventions, with designs aimed at reducing the duration of night shift workers' involvement in the study. This aligns with recent recommendations to finely tailor treatments to better suit the shift worker population Reynolds, Kyle, et al., 2023; Reynolds, Sweetman, et al., 2023).

Despite the attrition rate, the ITT approach clearly shows that the study is effective on two primary outcome measures (day‐ISI and day‐TST), for which the BT‐SWD group showed significant greater improvement than the control group with large ESs. Furthermore, the PP approach helps to underscore that BT‐SWD is effective on the same two outcome measures with large and important ESs for those who received the treatment. These results combined with the high treatment adherence rate and the absence of a SWD diagnosis after treatment indicate that night shift workers who received BT‐SWD had an excellent treatment response. The perceived insomnia severity was moderate or low before treatment, a phenomenon that might be attributed to the unique nature of daytime sleep. Indeed, an individual who sleeps during the day and experiences insomnia can still manage to participate in daily activities (e.g. seeing friends, going to the supermarket, etc.), activities that are often impractical at night in many cities where most establishments are closed during those hours. A similar ISI level of moderate insomnia severity with improvements after treatment in shift workers was previously reported (Jarnefelt et al., 2020; Peter et al., 2019). As reported by Reynolds, Sweetman, et al. (2023), the ISI demonstrated a decrease in other studies; however, it did not reach the established minimum clinical difference of six points. The improvement in insomnia severity obtained in the present study may be attributed to the treatment itself and to the methodology used to assess insomnia severity by separately capturing daytime and nighttime aspects, as participants completed the ISI twice.

The significant increase in daytime TST is an inestimable result for a behavioural intervention and partially confirms the first hypothesis. Moreover, this improvement is consistent with our previous pilot studies (Claveau et al., 2017; Vallières et al., 2015). Daytime sleep is likely to be the most difficult sleep period for shift workers (Boivin & Boudreau, 2014), and the target of hypnotic or chronotherapy for shift workers (Kalkanis et al., 2023). Until now, previous studies investigating CBT‐I for shift workers did not have significant results for sleep duration (Reynolds, Kyle, et al., 2023; Takano et al., 2023). Because previous studies did not differentiate between daytime and nighttime sleep, it is impossible to determine the specific type of sleep to which they were referring in their study or whether their sleep variables encompassed both. The results obtained on daytime TST in the present study may be attributed to the fact that BT‐SWD targets each sleep period and prevents stimulation when night shift workers follow stimulus control instructions during a sleep period. Our results support the notion that careful use of all sleep windows is needed while applying BT‐SWD as shift workers' sleep periods are likely interconnected as we previously proposed (Vallières et al., 2021). These results can also be attributed to the homogeneity of the sample in terms of night work schedules in the present study.

The present study shows that improving sleep helps to decrease the mental health problems experienced by shift workers as recently suggested by Reynolds et al. (2022). BT‐SWD had a positive impact on several insomnia‐related cognitive variables, such as intrusive thoughts at bedtime and pre‐sleep cognitive arousal, partially confirming our hypothesis. Moreover, BT‐SWD decreased psychological distress as results on treatment stability showed that depression and anxiety levels were improved after treatment. The mental health improvements observed are similar to other studies in CBT‐I for shift workers (Claveau et al., 2017; Dahlgren et al., 2022; Jarnefelt et al., 2012; Jarnefelt et al., 2020; Lee et al., 2014; Peter et al., 2019; Vallières et al., 2015). Even though most of these other studies did not show sleep duration improvements or clinical improvements in insomnia severity, they all demonstrated shift workers' satisfaction with treatment and/or some mental health improvement. Therefore, it seems that shift workers need psychosocial interventions to counteract shift work consequences.

The PP strategy analysis, incorporating the conservative Bonferroni correction, along with the stability of treatment efficacy analysis, highlight additional results that can contribute to enriching future studies. Indeed, these analyses indicate that daytime sleep latency and TWT improved with large ESs, and thus must be monitored in other studies. These variables are important for night shift workers as they have been previously identified as treatment targets for night shift workers with SWD (Vallières et al., 2021). Furthermore, the stability analysis showed that BT‐SWD has a positive effect on shift workers' night sleep decreasing TWT and SOL while increasing SE, the three variables identified as nearly significant by the PP approach. The effects obtained are similar to what is normally expected for CBT‐I in the general population (Maurer et al., 2021; Takano et al., 2023), and confirm our hypothesis. For night shift workers reversing sleep schedules on days off, consolidating nighttime sleep is likely to provide a better recovery before going back to a night shift. DBAS for night shift workers might also be a target in future studies evaluating CBT‐I efficacy for shift workers as the stability analysis showed that this score was decreased after BT‐SWD. This is aligned with at least one other study (Jarnefelt et al., 2020) reporting a decrease in DBAS after CBT‐I for shift workers.

A combination of treatments targeting all SWD contributors could be further developed. It could be hypothesised that positive and cumulating effects on sleep could be possible if night shift workers have adequate light exposure at night, wear blue‐light‐blocker glasses while commuting home, and follow BT‐SWD for their sleep periods. Further studies need to address the question of treatment combination given work schedule and type of work. In addition, because cognitive therapy is known to be effective in reducing insomnia symptoms (Harvey et al., 2014), and because shift workers present with negative ruminations accompanied by specific worries (Bastille‐Denis et al., 2020), a next step would be to adapt and test cognitive therapy added to BT‐SWD.

### 4.1 Strengths and limitations

The present study possesses several strengths that increase result reliability. First, a rigorous design providing a clear control to compare the evaluated treatment was used. In addition, the inclusion criteria provide a homogeneous sample of night shift workers in terms of work schedule and type of work. Third, the evaluation process using a clinical semi‐structured interview to diagnose SWD ensures that the treatment was delivered to people in need. Fourth, no adverse events were reported to the therapists during treatment and none of the participants had an unexpected effect or an increase in sleepiness following first treatment introduction. Fifth, the use of both ITT and PP strategies for statistical analyses enhances robustness of the obtained results. Nevertheless, the attrition rate as explained above is also an important limitation that can be explained by the design and selection criteria. Other limitations refer to the change of the waiting time duration. Although this change applied to only two participants, this might have influenced the post‐waiting scores obtained. Some other limitations are derived from the methodology. For example, participants were not screened for medical conditions by a general practitioner. The MINI relies on DSM‐IV criteria rather than the more current DSM‐5. Nevertheless, the MINI did enable the screening for exclusion criteria, and it was not utilised for screening for SWD and insomnia, which are the primary focus of BT‐SWD. Objective sleep was not assessed either. Polysomnography would have posed additional demands for night shift worker participants, while actigraphy could not be worn by all participants. Finally, results might not be generalisable to other populations of shift workers (e.g. firefighters) with other work schedules and environments different from the healthcare workers sampled.

## 5 CONCLUSIONS

The BT‐SWD is effective for improving insomnia severity and daytime sleep duration, and these improvements are significant, large and maintained over time. Furthermore, BT‐SWD improves anxiety and decreases cognitive activation before sleep and intrusive thoughts before bedtime. These positive effects of BT‐SWD might indicate that if cognitive therapy is added to BT‐SWD, it should target specific shift workers' worries rather than beliefs about sleep. Although the attrition rate was high, those who received the treatment greatly benefitted from it. An e‐health BT‐SWD would help circumvent attrition rate limitations observed in the present study and would allow a suitable adaptation of BT‐SWD within the shift work environment.

## AUTHOR CONTRIBUTIONS

**Annie Vallières:** Conceptualization; investigation; funding acquisition; writing – original draft; methodology; writing – review and editing; resources; visualization; supervision. **Alric Pappathomas:** Methodology; writing – review and editing. **Séverine de Billy Garnier:** Conceptualization; investigation; methodology; validation; project administration; visualization; writing – original draft. **Chantal Mérette:** Formal analysis; writing – review and editing; software; writing – original draft. **Julie Carrier:** Writing – review and editing; data curation. **Tyna Paquette:** Data curation; writing – review and editing. **Célyne H. Bastien:** Methodology; conceptualization; writing – review and editing.

## FUNDING INFORMATION

This work was funded by the Canadian Institutes of Health Research under Grant CIHR: MOP‐93254 awarded to Annie Vallières.

## CONFLICT OF INTEREST STATEMENT

Annie Vallières: financial disclosure: has grants from the Canadian Social Sciences and Humanities Research Council (SSHRC: #125553), the Canadian Institutes of Health Research (PJK‐179821), and the New Frontiers in Research Fund (#NFRFR‐2021‐00392) that are not related to the present study, and a researcher‐initiated grant from EISAI Inc. (New Jersey) not related to the present study; non‐financial disclosure: none. Alric Pappathomas: financial disclosure: none; non‐financial disclosure: none. Séverine de Billy Garnier: financial disclosure: none; non‐financial disclosure: none. Chantal Mérette: financial disclosure: has a grant from the CIHR (#341667), but it is not related to the present study; non‐financial disclosure: none. Julie Carrier: financial disclosure: has grants from the Canadian Institutes of Health Research (PJK‐179821) and the New Frontiers in Research Fund (#NFRFR‐2021‐00392) that are not related to the present study, and a researcher‐initiated grant from EISAI Inc. (New Jersey) not related to the present study; non‐financial disclosure: none. Tyna Paquette: financial disclosure: none; non‐financial disclosure: none. Célyne H. Bastien: financial disclosure: has grants from the Canadian Institutes of Health Research: PJK‐179821 and IE521323, from the New Frontier in Research Funds (#NFRFR‐2021‐00392), from the Brain Canada Foundation (PSG2019), and a researcher‐initiated grant from EISAI Inc. (New Jersey) not related to the present study; non‐financial disclosure: none.

## Data Availability

The data that support the findings of this study are available on request from the corresponding author. The data are not publicly available due to privacy or ethical restrictions.
